# Recurrent Deep Vein Thrombosis in a Middle-Aged Woman Due to Congenital Absence of the Inferior Vena Cava: A Case Report

**DOI:** 10.7759/cureus.100151

**Published:** 2025-12-26

**Authors:** Zachary R Pulliam, Goetz Kloecker

**Affiliations:** 1 Hematology and Medical Oncology, University of Louisville, Louisville, USA; 2 Hematology and Oncology, University of Louisville, Louisville, USA

**Keywords:** agenesis of the ivc, deep vein thrombosis (dvt), hypercoagulation, mechanical thrombectomy (mt), systemic anticoagulation

## Abstract

Deep venous thrombosis (DVT), either as an initial or recurrent event, is a common problem hematologists encounter while caring for hospital inpatients. It is caused by a variety of factors, sometimes anatomic in nature, and is managed with medical anticoagulation and, in some cases, procedural interventions such as mechanical thrombectomy. A 53-year-old woman, with a history of DVT of the lower extremity four years prior, presented to the emergency department with rapid development of swelling and pain in the left lower extremity after missing two doses of her anticoagulant. She was found to have an occlusive DVT in the left lower extremity requiring surgical thrombectomy. A computed tomography scan of the abdomen and pelvis showed the absence of the inferior vena cava with collaterals draining into the azygos vein. She had negative thrombophilia testing. The initial and recurrent thrombotic events were attributed to stagnant and turbulent blood flow due to anomalous venous drainage from the lower extremities.

## Introduction

Deep venous thrombosis (DVT) is a problem frequently encountered by hematologists. It is effectively treated with medical anticoagulation and sometimes requires surgical intervention. Recurrent DVT presents challenges, and the anticoagulation strategy is determined by etiology and the presence or absence of both modifiable and non-modifiable risk factors. Certain primary thrombophilic conditions can cause recurrent and breakthrough DVT, such as the factor V Leiden mutation, antiphospholipid syndrome, the prothrombin 20210 mutation, and antithrombin deficiency. Other provoking risk factors can include malignancy and trauma [1].

For patients who have previously developed a DVT, noncompliance with the prescribed anticoagulant can cause recurrence of thrombosis. Rarely, congenital vascular anomalies disrupt normal venous return, which can predispose to DVT. Congenital absence of the inferior vena cava (IVC) has previously been described as a risk factor for unprovoked DVT, particularly in patients under 30 years old and with a male predominance [2]. The case presented here highlights an unusual case of congenital agenesis of the IVC causing an initial unprovoked lower extremity DVT in a middle-aged woman, with recurrence after a very brief interruption of anticoagulation.

## Case presentation

A relatively healthy 53-year-old woman presented with acute-onset left lower extremity swelling and pain at a hospital along her long-haul trucking route. She had a history of a left lower extremity DVT, diagnosed four years prior, and hypothyroidism. The patient had run out of her rivaroxaban, which she was prescribed for her prior DVT event, two days prior to the development of the symptoms. She noted that she had previously been told that she had abnormal blood vessels, but was uncertain what the abnormality was. She reported no family history of blood clots, bleeding disorders, or malignancy. She did not use alcohol, tobacco, or illicit drugs.

Her physical exam was significant for severe left lower extremity edema with dermal tightness, tenderness to palpation over the left calf, and tenderness of the left lower extremity with passive dorsiflexion of the left ankle. Her vital signs were stable at the time of initial presentation. 

**Figure 1:**
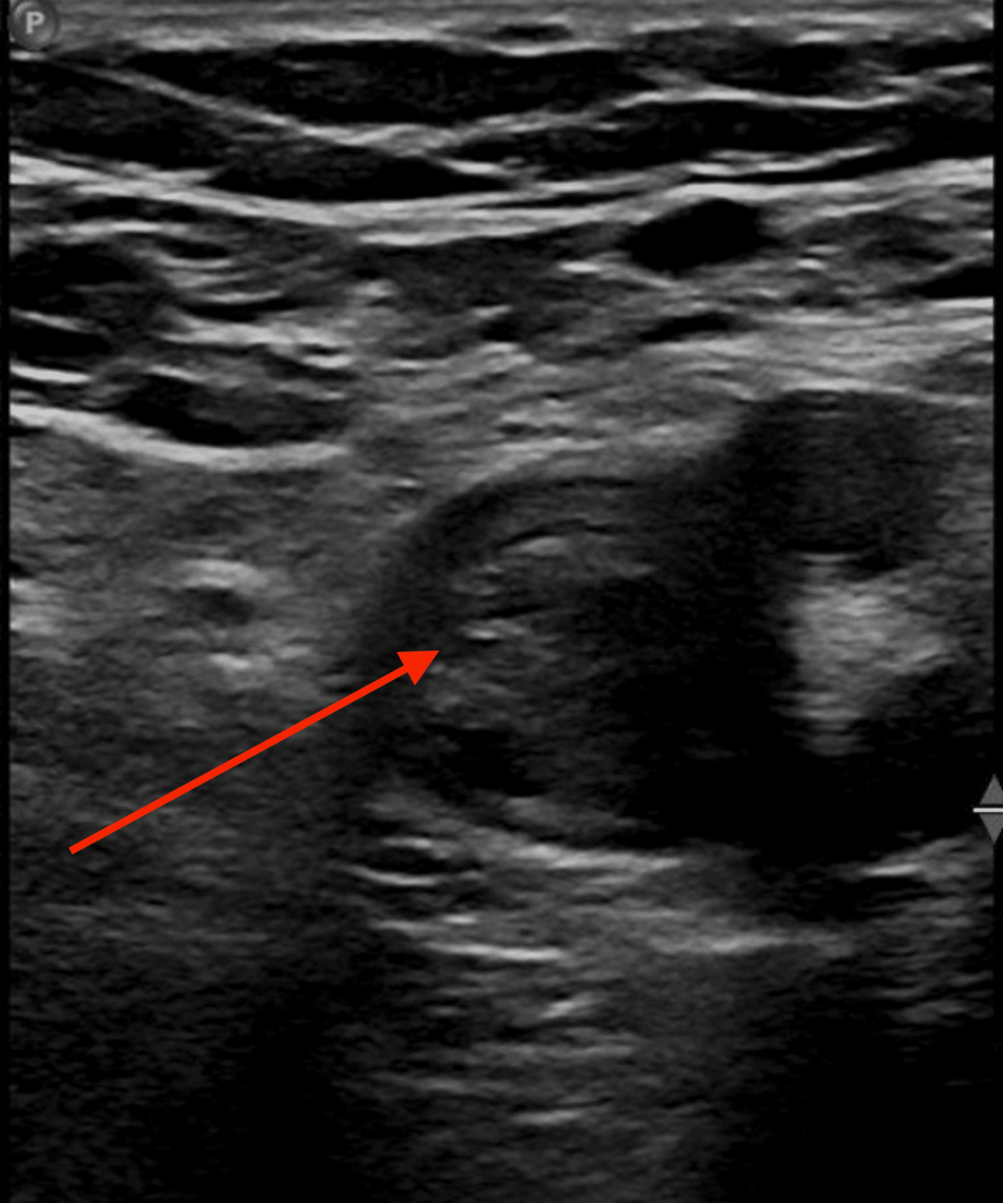
Ultrasound of the left common femoral vein demonstrating a large deep vein thrombosis Red Arrow: noncompressible thrombus in the left common femoral vein

**Figure 2:**
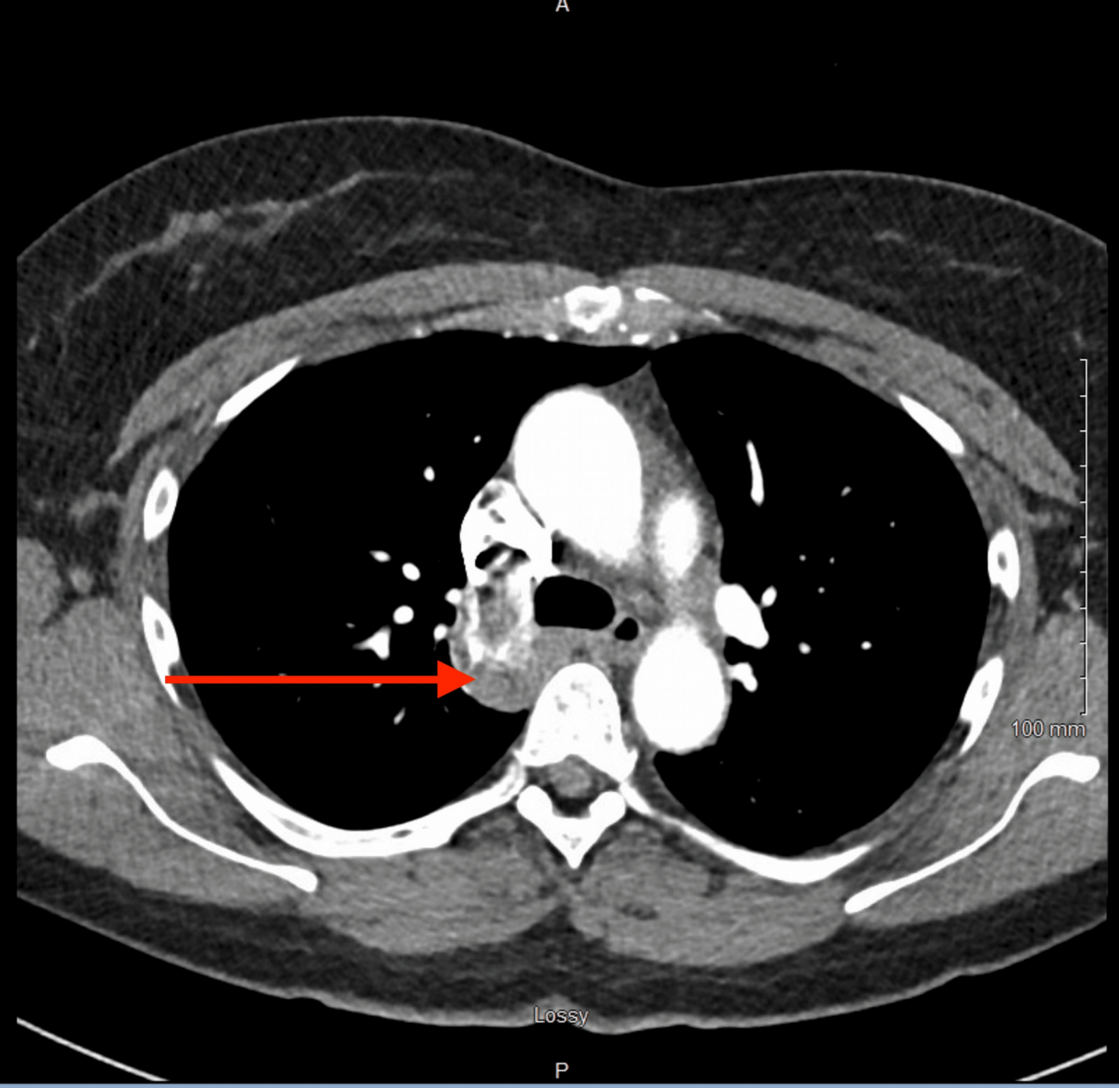
CT (pulmonary embolism protocol) showing an enlarged azygos vein (red arrow) in the thorax

Duplex ultrasonography of the lower extremities was obtained, which demonstrated extensive DVT in the left lower extremity extending from the femoral vein to the calf veins (Figure 1). A computed tomography (CT) scan of the chest with pulmonary embolism (PE) protocol demonstrated an enlarged azygous vein extending into the abdomen without any evidence of PE, thoracic aortic pathology, or parenchymal lung pathology (Figure 2). 

Complete metabolic panel and complete blood count were normal, with the exception of mild anemia with a hemoglobin of 11.7 g/dL. The patient additionally reported that she had previously been tested for thrombophilic conditions, and review of outside facility records confirmed negative comprehensive thrombophilia testing, including Factor V Leiden, prothrombin mutation, antiphospholipid syndrome testing, proteins C and S deficiencies, and antithrombin III deficiency. 

**Figure 3:**
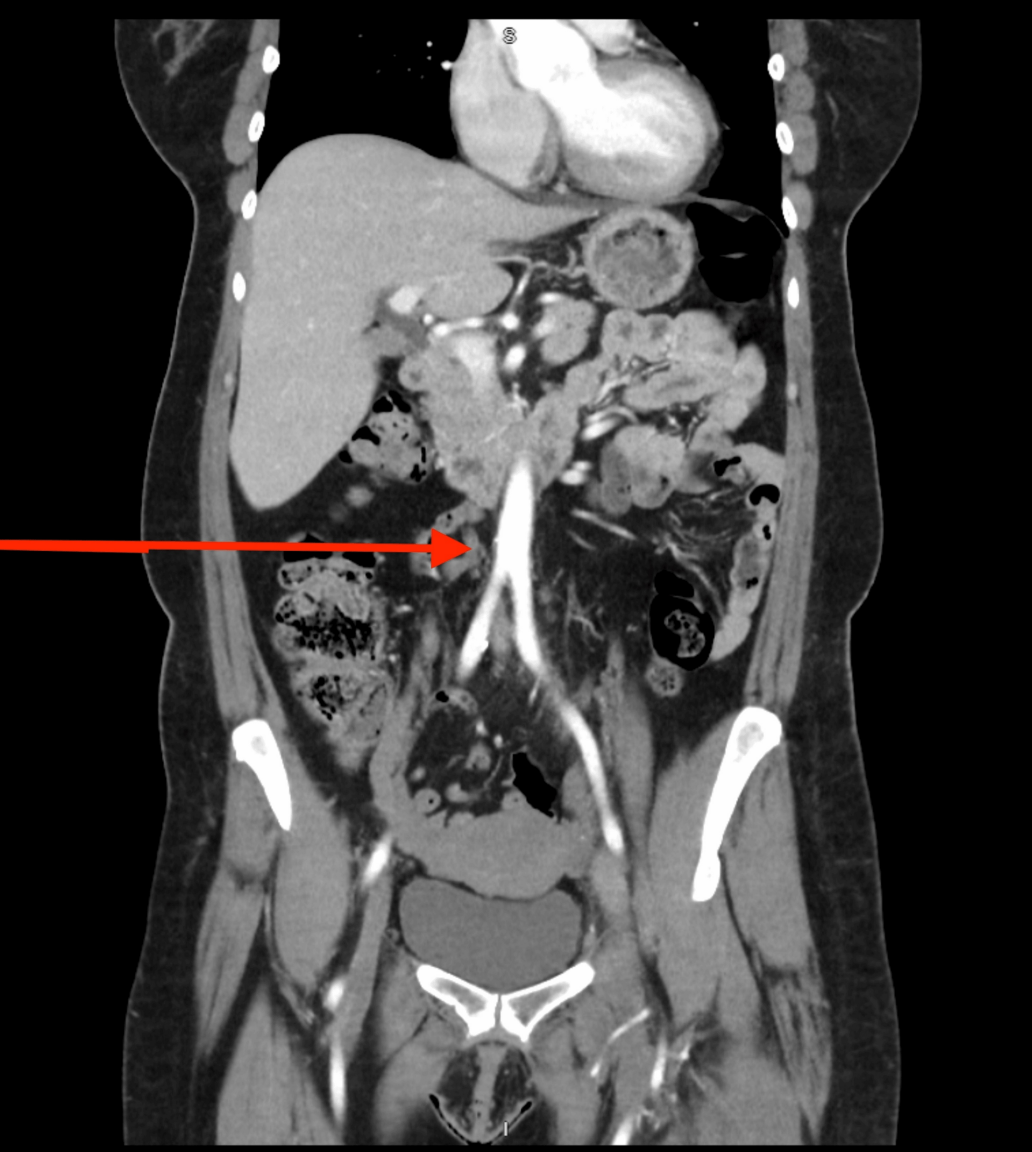
CT abdomen and pelvis showing absence of inferior vena cava in the abdomen (red arrow)

A CT of the abdomen and pelvis with contrast (Figure 3) was obtained to assess for anomalous vasculature, which she had alluded to, in addition to further characterizing the azygous vein abnormality noted on the earlier CT (PE). This study redemonstrated the left iliofemoral deep venous thrombus extending to the most inferior slices of the imaging, as well as the absence of the IVC with numerous retroperitoneal collateral veins entering the azygous circulation, leading to an upstream enlargement of the azygous vein.

The patient was started on a heparin infusion for anticoagulation and was evaluated by vascular surgery, who took her for percutaneous transluminal venous thrombectomy of the left lower extremity, with operative findings of an acute-on-chronic DVT. A mechanical thrombectomy was performed through a 16 French lightning catheter, followed by balloon angioplasty with a 12 mm x 4 cm high-pressure balloon of a tight stenosis in the left common iliac vein with stenting using a 20 mm x 80 mm wallstent. Post-dilation was performed with a 14 mm x 4 cm high-pressure balloon, and further mechanical thrombectomy was performed, with venography showing patent vessels thereafter. She experienced rapid and dramatic improvement in her symptoms after mechanical thrombectomy and was transitioned from infusional heparin back to rivaroxaban for outpatient anticoagulation. She was discharged on postoperative day 2.

## Discussion

DVT is a common problem causing hospital admission that hematologists are frequently consulted for recommendations regarding etiology and anticoagulation management. Thrombophilic conditions, including antiphospholipid syndrome, Factor V Leiden mutation, protein C deficiency, amongst a handful of others, are primary hematologic issues that predispose individuals to clotting events. Additional risk factors for venous thromboembolic events include underlying malignancy, trauma, mechanical compression of the vessels, and a variety of other causes. Conceptually, the pathophysiology of venous thrombosis development as taught in medical schools by the principles of Virchow Triad, which consists of endothelial injury, venous stasis, and hypercoagulable states [3].

Congenital absence of the IVC is a rare anatomic anomaly that is only seen in about 0.0005% and 1% of the population [4]. In young patients with otherwise unexplained deep vein thrombosis, it can be present in up to 5% of cases [5]. One can easily and rationally conceptualize that the absence of this large caliber vessel would cause turbulence and relatively static venous return to the heart, predisposing to thrombotic events as part of a virtual triad as mentioned above. Interestingly, our patient had never experienced DVT symptoms prior to her first event four years prior to this presentation. She had adapted to the absence of the IVC through the development of large collateral venous structures that drained into the azygous vein to reach the central circulation. Azygos continuation of the IVC in the setting of congenital agenesis of the IVC has previously been reported in other cases [6]. The employment of the present patient as a long-haul truck driver caused further venous stasis on top of that posed by her anatomic anomaly and interruption in anticoagulation, which led to the rapid development of recurrent DVT.

The risk of recurrent DVT in patients with an unprovoked clot after three months of anticoagulation, when anticoagulation is stopped, is about 10% in the first year [7]. Risk factors for recurrent DVT include male sex, presence of both DVT and PE at the time of the initial event, and persistent provoking risk factors [7]. We hypothesize that the patient suffered from a rapid recurrence of DVT due to her underlying IVC agenesis as a non-modifiable provoking risk factor, especially in light of the absence of other thrombophilic conditions. Our recommendation for management of these patients is advising indefinite anticoagulation in consideration of a non-modifiable risk factor, as has been suggested by a previous retrospective, observational study, which demonstrated lower recurrent DVT risk in patients with IVC agenesis on indefinite anticoagulation [8].

Recommendations surrounding the duration of anticoagulation are generally made based on the presence of provoking and/or modifiable risk factors for thrombotic events [9]. In the absence of CT imaging demonstrating the vascular anomalies in this patient, a clinician could easily attribute her recurrent clot to immobility from working as a long-haul truck driver and potentially only recommend a short, definite duration of anticoagulation of three to six months. This case highlights the importance and rarity of anatomic abnormalities causing a non-modifiable risk factor for recurrent thromboses in a patient who had not suffered a thrombotic event prior to middle age, thus indicating indefinite anticoagulation to prevent recurrence. Given the rarity of this condition, the practicality of pursuing evaluation for vascular anomalies in patients with recurrent DVT is uncertain and represents an issue to be explored with further research. 

## Conclusions

Congenital absence of the IVC can be a cause of recurrent unprovoked DVT in young patients, and can, in rare cases, be a cause of unprovoked thrombotic events in older patients who did not have their first event until middle-age. 
